# The role of salivary contents and modern technologies in the remineralization of dental enamel: a narrative review

**DOI:** 10.12688/f1000research.22499.2

**Published:** 2020-04-01

**Authors:** Imran Farooq, Amr Bugshan

**Affiliations:** 1 Department of Biomedical Dental Sciences, College of Dentistry, Imam Abdulrahman Bin Faisal University, Dammam, 31441, Saudi Arabia

**Keywords:** Saliva, Enamel, Remineralization, Fluoride, Calcium Phosphate

## Abstract

Human enamel once formed cannot be biologically repaired or replaced. Saliva has a significant role in remineralization of dental enamel. It not only has a buffering capacity to neutralize the oral cavity’s low pH generated after acidic encounters, but also acts as a carrier of essential ions, such as fluoride, calcium and phosphate, which have a positive role in enamel’s remineralization. This review discusses how salivary contents, like proteins and enzymes, have a natural role in enamel’s mineralization. In addition, the presence of ions, such as fluoride, calcium and phosphate, in saliva further enhances its capability to remineralize the demineralized enamel surface. The review further examines modern innovative technologies, based on biomimetic regeneration systems, including dentin phosphoproteins, aspartate-serine-serine, recombinant porcine amelogenin, leucine-rich amelogenin peptide and nano-hydroxyapatite, that promote enamel remineralization. Fluoride boosters like calcium phosphates, polyphosphates, and certain natural products can also play an important role in enamel remineralization.

## Introduction

Dental enamel is a calcified tissue that forms the outer protective covering of the anatomical crown of a tooth ^1^. Enamel once formed cannot be biologically repaired or replaced ^2^. The oral cavity continuously goes through cycles of demineralization and remineralization ^3^. Loosing minerals from the tooth after an acidic encounter is called demineralization, whereas restoration of these minerals back into the tooth structure is called remineralization ^4^. During demineralization, the enamel surface becomes rough and rugged upon acidic encounter. Thus throughout the life of a tooth, there are enamel demineralization/remineralization cycles that dictate the extent of mineral balance and tissue integrity or degradation ^3^. Human saliva has a buffering role and acts as a carrier of essential ions that can bring a constructive change in the structure of enamel, promoting remineralization ^5^.

**Figure 1.**
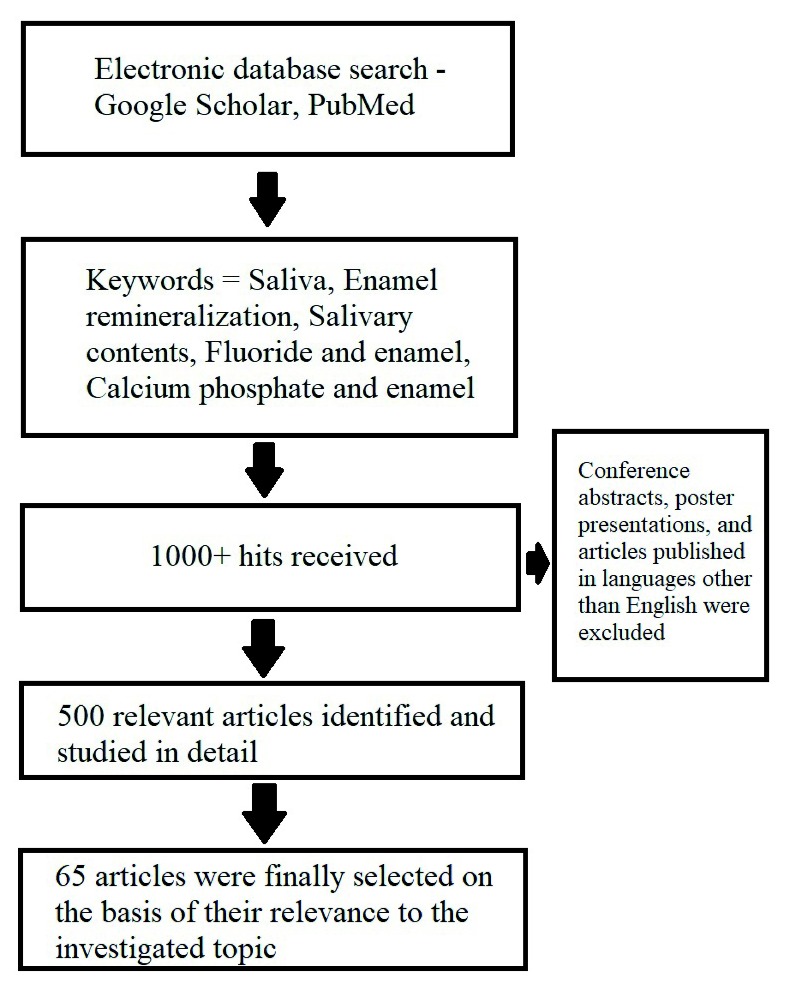
Flow chart depicting search strategy included in this article.

This review is aimed at providing an overview of enamel structure and an in-depth insight on its mineralization mechanism carried out by salivary contents. The summary of search strategy involved in this study is shown in Figure 1.

## Structure of dental enamel

Dental enamel is composed of 96% inorganic material, 3% water, and 1% organic matrix ^6^. The inorganic component of dental enamel is hydroxyapatite (HAP) crystal and human enamel is a hard, acellular, and avascular tissue ^1^. Being acellular, it cannot be automatically replaced or repaired if damaged ^2^. However, its very highly mineralized nature makes it extremely resistant to destruction ^7^. Enamel’s high mineral content also makes it susceptible to demineralization by acids formed by bacteria in the mouth, which leads to dental caries ^3^.

Tooth enamel is an intricate structure and requires a keen sense of three-dimensional geometry to appreciate it. Early micro-anatomical descriptions are incomplete and reflected the restrictions of light microscopy. Only after the advent of modern techniques using transmission and scanning electron microscopy, the more complex structure of enamel was revealed. Enamel’s composition includes fiber-like mineralized crystals and a small proportion of water and proteins that clamp the mineralized fibers with each other ^8^. This arrangement of mineralized and non-mineralized components of enamel, disperses the forces passing through teeth and in this manner shields them from fractures ^4^. Enamel is composed of discrete basic units called Enamel Rods (formerly enamel prisms), each surrounded by a rod sheath with these packed together and embedded in an inter-rod (inter-prismatic) substance ^9^. Ameloblasts elaborate and secrete the enamel protein matrix that is subsequently mineralized by the addition of calcium phosphate crystallites ^10^. When fully differentiated, ameloblasts are tall columnar cells fully equipped for protein synthesis ^11^. The secretory end of the cell develops a projection (Tome’s process) through which the protein matrix and crystallites are laid down ^12^. The appearance of the inter-rod (inter-prismatic) substance and rod sheath at a light microscope level is due to the changes in crystallite orientation as they are laid down by ameloblasts ^13^. The main body of the enamel rod is ~5µm in diameter but towards the enamel surface, the diameter is greater ^14^. The number of ameloblasts that form the enamel are thought to remain constant for each tooth ^15^. At the beginning and end of amelogenesis, Tome’s process is absent at the secretory end of ameloblasts and unstructured enamel (rod-less/prism-less) is produced ^16^. This prism-less enamel varies in thickness but there is a general agreement that prism-less enamel is composed of HAP crystals that are arranged parallel to one another and perpendicular to the enamel’s surface ^17^.

It is quite a well-established fact that fluoride helps in the prevention of dental caries ^18^. Fluoride has remained under extensive investigations because of its effects on the structure and properties of tooth minerals ^19^. Ionic substitution is a common phenomenon in the oral cavity and two major stake holders are enamel and saliva ^20^. The carbonate ion can replace hydroxyl or phosphate ions, magnesium can replace calcium, and fluoride can replace hydroxyl ions in the crystal lattice ^3^. These ionic substitutions have a significant influence on the behavior of apatite including its solubility ^21^. It is well-known that fluoride in the saliva (when it comes in contact with enamel) replaces the hydroxyl ion in the apatite crystal structure thus changing HAP into fluorapatite, which is more resistant to acidic attacks ^22^. Therefore, understanding human body glands, particularly salivary glands, prior to going into details on the influence of salivary contents on remineralization of enamel becomes necessary. 

## Classification of glands and types of salivary glands

A gland is an organ that synthesizes a secretion for release and two major types of glands found in the human body are exocrine and endocrine glands ^23^. Exocrine glands are those which lack a duct system and secrete their products through basal lamina into the bloodstream to regulate the body (for example, salivary and sweat glands) ^24^. Endocrine glands possess a duct system and discharge their products into the ducts, which then lead them into the outer environment (for example, pituitary, thyroid and adrenal glands) ^24^. A salivary gland is an organ that releases a secretion in the oral cavity, and it is further classified into major and minor types ^25^. Major salivary glands are situated at a distance from the oral mucosa but are connected to it through extra glandular ducts ^26^. Minor salivary glands reside in the mucosa or sub mucosa and can open directly inside the oral cavity ^27^. The three major paired salivary glands in humans are parotid, submandibular, and sublingual glands ^28^. Among the minor salivary glands, the important ones are von Ebner, Weber, buccal, labial, and palatal glands ^29^.

## Role of saliva and its contents in remineralization of dental enamel

Human saliva is comprised of numerous contents and therefore has various functions. Saliva is a fluid that protects the mouth against harmful microorganisms and irritants ^30^. It not only lubricates the oral tissues but also helps in various other functions, such as speech, mastication and swallowing, as well as protection of the teeth and oral tissues ^31^. The ability of saliva to remineralize tooth enamel is dependent on various factors, discussed below.

### Buffering capacity

The buffering capacity of saliva displays an imperative role in maintaining the level of pH in both saliva and plaque, therefore helping in neutralizing the effects of acid exposure ^5^. The three buffer systems present in the saliva are carbonic acid/bicarbonate system (the most important), phosphate system, and protein system ^32^. The breakdown of proteins by bacterial originated urease to urea and ammonia aids plays a role in maintaining a neutral pH in oral cavity ^33^. The intake of certain supplemental hormones, like estrogen and progesterone, could also lead to an improvement in salivary buffering capacity in individuals ^33^. 

### Salivary proteins

Proteins are part of the normal anatomy of human saliva and some salivary proteins, such as proline rich proteins, statherin and histatins, have an affinity for enamel surfaces and thus help remineralization by increasing local calcium concentration ^34^. Other proteins, e.g. cathelicidin LL3, have an antimicrobial function; histatins are antibacterial, and alpha-defensin HNP1–3 are antiviral in function ^35^. Certain proteins, like lactoferrin, can prevent *Streptococcus mutans* growth, as they can isolate iron from the oral environment, which is vital for bacterial metabolism ^33, 36^.

### Salivary enzymes

Lysozyme enzyme is found in humans in serum, amniotic fluid, and saliva ^37^. Lysozyme found in saliva helps in the lysis of bacterial cells ^31 ^ and is especially potent against gram-positive bacteria ^38^. Lysozyme is also believed to have a part in prevention of bacterial aggregation and adherence, thus providing an opportunity for bacterial autolysins, which can then destroy cell walls of bacteria ^39^. Salivary peroxidase enzyme preserves oral health by preventing build-up of hydrogen peroxide and deactivates carcinogenic compounds ^37^.

### Salivary fluoride ions

**Figure 2.**
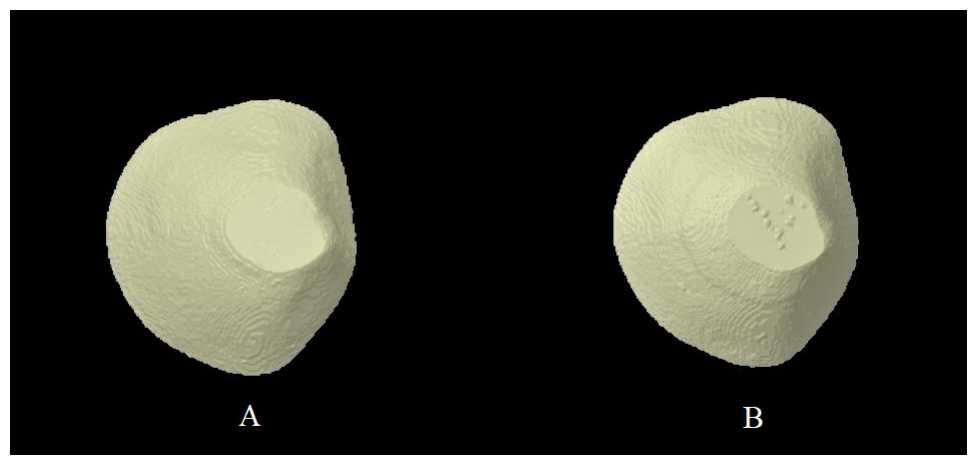
Micro-computed tomography (micro-CT) image of enamel surface **A**) Ground and polished **B**) Remineralized enamel surface after 24 hours of exposure to a mixture of fluoride toothpaste and distilled water (Courtesy Dr. Saqib Ali, College of Dentistry, Imam Abdulrahman Bin Faisal University, Dammam, Saudi Arabia).

At a normal pH, saliva is supersaturated with calcium and phosphate ions, therefore, demineralization does not take place ^40^. Acids of bacterial origin and those coming from food or drinks tend to shift the equilibrium towards the mineral loss ^3^. The phosphate concentration of saliva is reduced and at pH 5.5, saliva no longer remains supersaturated and demineralization initiates ^5^. Saliva acts as a remineralizing agent and as a delivery vehicle of ions, like fluoride, which can then incorporate in the tissues ^41^. It should be noted however, that complete substitution of fluoride with hydroxyl does not occur, but even a limited replacement is able to significantly reduce the incidence of dental caries and initiate remineralization ^33^. Remineralization of dental enamel surface after being exposed to a solution containing mixture of fluoride toothpaste and distilled water is shown in Figure 2. The presence of partially demineralized crystallites is a pre-requisite for remineralization as these crystallites then act as nuclei for mineral deposition ^42^. Fluoride in saliva could have three major roles; prevention of demineralization, promotion of remineralization, and interfering with growth of bacteria ^43^. Dental caries starts with a white spot lesion (WSL) and these lesions can be reversed with appropriate fluoride therapy ^44^. However recently, Dai *et al.* have reported that only fluoride therapy (FT) could be insufficient for reversal of WSLs and a combination of FT with fluoride varnish and FT with casein phosphopeptide-amorphous calcium phosphate (CPP-ACP) application could be more effective in remineralization ^45^.

### Salivary calcium and phosphate ions

Both calcium and phosphate ions are required by fluoride to promote the natural remineralization process of enamel ^46^. The water content present in enamel facilitates influx of acids and efflux of minerals thus causing demineralization ^20^. It has been previously reported that during the demineralization process, calcium is released before phosphate ions; therefore using a calcium-based product could suppress the demineralization process effectively ^5^. Due to the use of increased amounts of fluoride in dentifrices, concerns regarding its toxicity were raised and casein phosphopeptides (calcium and phosphate-based products) have been introduced ^47^. For an equal rate of supersaturation, an ideal rate of enamel remineralization can be attained with a calcium/phosphate (Ca/P) ratio of 1.6 ^48^. In the plaque, the Ca/P ratio is ~0.3, so supplemental calcium will enhance remineralization of enamel ^48^. Salivary pellicle starts forming on the tooth surface almost immediately post-absorption of proteins and peptides onto the enamel’s surface ^49^. Calcium binding peptides attract free calcium ions in the saliva and thus act as a pool for calcium ions in the pellicle ^50^. In addition, diffusion of calcium ions through the pellicle inside the enamel’s surface takes place easily and this regulates the remineralization process ^51^. Calcium phosphate embedded in salivary pellicle has a high solubility (almost ten times more than calcium phosphate in tooth mineral); therefore, it serves as a sacrificial mineral post-acidic challenge instead of calcium phosphate present in the tooth structure, preventing demineralization ^52^.

## Recent advances in enamel remineralization therapies

There have been many recent innovative advances in systems that act through saliva to promote enamel remineralization, but that do not depend on fluoride therapies ^53^. Philip divided these systems into two categories: (i) biomimetic regeneration technologies; and (ii) systems that boost fluoride effectiveness ^53^. In the first category, the most important is tooth regeneration via dentin phosphoprotein (DPP). It has been shown previously that DPP has the ability to remineralize the tooth surface when it is present in a solution containing calcium and phosphate, just like saliva ^54^. Many new systems have been derived based on DPP and among them the most active in promoting remineralization is aspartate-serine-serine (8DSS) ^55^. Application of 8DSS to the enamel surface does not only prevent leaching of ions from the enamel surface, but also promotes binding of calcium and phosphate ions from the saliva ^53^. Amelogenin is an important protein that regulates the growth and maturation of enamel crystals in newly formed enamel matrix ^15^. This protein is absent in mature enamel, meaning it cannot regenerate ^56^. Modern systems, such as recombinant porcine amelogenin (rP172) and leucine-rich amelogenin peptide, stabilize calcium phosphate to enhance crystal formation and direct mineral growth respectively ^57, 58^. Nanohydroxyapatite is another bioactive material that can promote enamel remineralization ^53^. As these particles are very small (nano-sized), they bind strongly to the enamel surface and fill up the gaps and holes in the enamel surface to repair it ^53, 59^.

In the second category, which are also known as fluoride promoters, many modern systems are available ^53^. The most significant are calcium phosphate based systems and among them, the most important is CPP-ACP ^60^. CPP-ACP particles are readily soluble in saliva and thus localize in plaque and act as a reservoir of calcium and phosphate ions ^61^. Upon an acidic encounter, they release these ions to promote remineralization and inhibit demineralization ^61^. Another material to boost enamel remineralization is bioactive glass, which is based on calcium sodium phosphosilicate composition ^62^. It dissolves in aqueous solution to release sodium, calcium, and phosphate ions in the saliva, to which they interact, leading to deposition of a layer of HAP on the enamel’s surface ^63^. Another major system in the second category is polyphosphate-based systems and among them the most essential is sodium trimetaphosphate (STMP) ^64^. STMP not only promotes remineralization, but also inhibits its demineralization ^64^. Some other natural products, such as thymoquinone, have shown good capability in promoting enamel remineralization *in vitro* ^65^, but data on its *in vivo* effectiveness is still anticipated ^65^.

Enamel remineralization is vital for a tooth as it lacks the capability to regenerate it. More research on remineralizing materials is required as it would significantly decrease incidence of dental caries.

## Conclusion

Saliva contains many important substances and also acts as a transporter of many important ions, such as calcium, phosphate and fluoride, which are essential for the promotion of remineralization. Pathogenicity of dental erosion and caries is directly influenced by the buffering capacity and contents of saliva. Saliva helps to maintain a constant reservoir of ions that help to neutralize the pH and prevent demineralization. Modern innovative technologies, for example biomimetic regeneration technologies, including dentin phosphoproteins, aspartate-serine-serine, recombinant porcine amelogenin, leucine-rich amelogenin peptide and nano-hydroxyapatite, promote enamel remineralization. Fluoride boosters, like calcium phosphates, polyphosphates and natural products, also play an important role in enamel remineralization.

## Data availability

No data are associated with this article.
