# Supplementary material for: The role of salivary contents and modern technologies in the remineralization of dental enamel: a narrative review
Source: F1000Res. 2021 Jun 15;9:171. Originally published 2020 Mar 9. [Version 3] doi: 10.12688/f1000research.22499.3 (PMC7076334; doi:10.12688/f1000research.22499.3)
Supplement: Peer review history

# Peer review: Reviewer response for version 3

**DOI:** 10.5256/f1000research.57771.r87619

**Published:** 2021-06-16

**Authors:** A. Thirumal Raj

**Affiliations:** 1 Department of Oral Pathology and Microbiology, Sri Venkateswara Dental College and Hospital, Chennai, Tamil Nadu, India

I have no further comments to make.

Is the review written in accessible language?

Yes

Are all factual statements correct and adequately supported by citations?

Yes

Are the conclusions drawn appropriate in the context of the current research literature?

Yes

Is the topic of the review discussed comprehensively in the context of the current literature?

Yes

Reviewer Expertise:

Oral pathology and microbiology

I confirm that I have read this submission and believe that I have an appropriate level of expertise to confirm that it is of an acceptable scientific standard.

# Peer review: Reviewer response for version 2

**DOI:** 10.5256/f1000research.25484.r61912

**Published:** 2020-04-14

**Authors:** Rizwan Ullah

**Affiliations:** 1 Department of Oral Biology, Sindh Institute of Oral Health Sciences, Jinnah Sindh Medical University, Karachi, Pakistan

Is the review written in accessible language?

Yes

Are all factual statements correct and adequately supported by citations?

Yes

Are the conclusions drawn appropriate in the context of the current research literature?

Yes

Is the topic of the review discussed comprehensively in the context of the current literature?

Yes

Reviewer Expertise:

NA

I confirm that I have read this submission and believe that I have an appropriate level of expertise to confirm that it is of an acceptable scientific standard.

# Peer review: Reviewer response for version 2

**DOI:** 10.5256/f1000research.25484.r61910

**Published:** 2020-04-03

**Authors:** Shakeel Abbas Kazmi

**Affiliations:** 1 Dundee Dental School, University of Dundee, Dundee, UK

The issues were addressed properly.

Is the review written in accessible language?

Yes

Are all factual statements correct and adequately supported by citations?

Yes

Are the conclusions drawn appropriate in the context of the current research literature?

Yes

Is the topic of the review discussed comprehensively in the context of the current literature?

Yes

Reviewer Expertise:

NA

I confirm that I have read this submission and believe that I have an appropriate level of expertise to confirm that it is of an acceptable scientific standard.

# Peer review: Reviewer response for version 2

**DOI:** 10.5256/f1000research.25484.r61911

**Published:** 2020-04-02

**Authors:** A. Thirumal Raj

**Affiliations:** 1 Department of Oral Pathology and Microbiology, Sri Venkateswara Dental College and Hospital, Chennai, Tamil Nadu, India

All the revisions implemented are to my satisfaction. I have no further comments to make.

Is the review written in accessible language?

Yes

Are all factual statements correct and adequately supported by citations?

Yes

Are the conclusions drawn appropriate in the context of the current research literature?

Yes

Is the topic of the review discussed comprehensively in the context of the current literature?

Yes

Reviewer Expertise:

Oral pathology and microbiology

I confirm that I have read this submission and believe that I have an appropriate level of expertise to confirm that it is of an acceptable scientific standard.

# Peer review: Reviewer response for version 1

**DOI:** 10.5256/f1000research.24830.r61099

**Published:** 2020-03-25

**Authors:** Shakeel Abbas Kazmi

**Affiliations:** 1 Dundee Dental School, University of Dundee, Dundee, UK

It is a well-written article. The role of salivary contents are comprehensively explained. I would like to suggest the following suggestions:

1. The inclusion criteria of the articles is a bit confusing. How the research started is well-written but later it is a bit confusing how 500 relevant articles were selected. It would be nice to know how these 500 articles were selected.
2. Some flow charts, for the selection of the articles for research can be helpful.
3. Similarly, if there could be a chart, diagram or table before the conclusion, it would help in learning about the article summary.

Is the review written in accessible language?

Yes

Are all factual statements correct and adequately supported by citations?

Yes

Are the conclusions drawn appropriate in the context of the current research literature?

Yes

Is the topic of the review discussed comprehensively in the context of the current literature?

Yes

Reviewer Expertise:

NA

I confirm that I have read this submission and believe that I have an appropriate level of expertise to confirm that it is of an acceptable scientific standard.

# Response

**Published:** 2020-03-27

**Authors:** Imran Farooq

**Affiliations:** College of Dentistry, Imam Abdulrahman Bin Faisal University, Dammam, Saudi Arabia

Thank you for your comments and accepting our article. We have revised the article as per your suggestions. 

1) We have added a flow chart depicting search strategy involved in this article. We hope this will answer your concern.

2) A flow chart has been added as per your suggestion.

3) Instead of a chart, we have added a micro-CT figure which shows remineralization of enamel surface.

# Peer review: Reviewer response for version 1

**DOI:** 10.5256/f1000research.24830.r61100

**Published:** 2020-03-16

**Authors:** A. Thirumal Raj

**Affiliations:** 1 Department of Oral Pathology and Microbiology, Sri Venkateswara Dental College and Hospital, Chennai, Tamil Nadu, India

It is a well executed review. There are a few suggestions which could improve the overall impact of the review: 

1. Adding a schematic summarizing the de-mineralization and re-mineralization of dental enamel would improve the overall impact of the article.
2. Was this a systematic review? If not mention that it is a narrative review based on published literature.
3. A table summarizing the various recent techniques in re-mineralization along with each of their advantages and limitations would be vital for the article.
4. The article seems to end abruptly. Kindly add a couple of sentences on prospects of further research as the concluding sentence.

The above-mentioned corrections are just suggestions to improve the overall quality of the article. I recommend the authors consider implementing the recommendations.

Is the review written in accessible language?

Yes

Are all factual statements correct and adequately supported by citations?

Yes

Are the conclusions drawn appropriate in the context of the current research literature?

Yes

Is the topic of the review discussed comprehensively in the context of the current literature?

Yes

Reviewer Expertise:

Oral pathology and microbiology

I confirm that I have read this submission and believe that I have an appropriate level of expertise to confirm that it is of an acceptable scientific standard.

# Response

**Published:** 2020-03-27

**Authors:** Imran Farooq

**Affiliations:** College of Dentistry, Imam Abdulrahman Bin Faisal University, Dammam, Saudi Arabia

Thank you for your comments and accepting our article. We have revised the article as per your suggestions. 

1) We have added a micro-CT image which shows remineralization of enamel's surface. 

2) We have now added the word "narrative" to the title.

3) This would be part of our next paper which would be dealing with this point. 

4) We have added few sentences to answer your concern.

# Peer review: Reviewer response for version 1

**DOI:** 10.5256/f1000research.24830.r61101

**Published:** 2020-03-13

**Authors:** Rizwan Ullah

**Affiliations:** 1 Department of Oral Biology, Sindh Institute of Oral Health Sciences, Jinnah Sindh Medical University, Karachi, Pakistan

I have the following comments and suggestions for the authors:

1. What is the reason for choosing 35 years and two databases Google Scholar and PubMed? As some of the studies that may be some important papers published in journals indexed in other databases will be missing.
2. Present complete article search and selection process in a flow chart outlining the exclusion of articles at each stage. In methodology give a brief account how it was decided to include or exclude an article.
3. Kindly rephrase the sentence on Page 2. " Only after the advent of modern techniques using transmission and scanning electron microscopy, was the more complex structure of enamel revealed".
4. Kindly rephrase the students on Page 3 "The breakdown of proteins by bacterial originated urease to urea and ammonia aids plays a role in maintaining a neutral pH in oral cavity"

Is the review written in accessible language?

Yes

Are all factual statements correct and adequately supported by citations?

Yes

Are the conclusions drawn appropriate in the context of the current research literature?

Yes

Is the topic of the review discussed comprehensively in the context of the current literature?

Yes

Reviewer Expertise:

NA

I confirm that I have read this submission and believe that I have an appropriate level of expertise to confirm that it is of an acceptable scientific standard.

# Response

**Published:** 2020-03-27

**Authors:** Imran Farooq

**Affiliations:** College of Dentistry, Imam Abdulrahman Bin Faisal University, Dammam, Saudi Arabia

Thank you for your comments and accepting our article. We have revised the article as per your suggestions. 

1) Many journals now consider references which are 10 years old outdated. We kept a bigger window for our article and this is the reason we did not include any reference which was published more than 35 years ago. To make sure we don't miss any important studies, we chose two databases instead of one.  

2) A flow chart has now been added which depicts the search strategy involved in this study.

3) This sentence has been rephrased.

4) We believe the sentence is clear and unfortunately, rephrasing it would make it unclear.
